# Metabolites of Purine Nucleoside Phosphorylase (NP) in Serum Have the Potential to Delineate Pancreatic Adenocarcinoma

**DOI:** 10.1371/journal.pone.0017177

**Published:** 2011-03-23

**Authors:** Shaiju K. Vareed, Vadiraja B. Bhat, Christopher Thompson, Vihas T. Vasu, Damian Fermin, Hyungwon Choi, Chad J. Creighton, Sitaram Gayatri, Ling Lan, Nagireddy Putluri, Gagan Singh Thangjam, Punit Kaur, Mohsen Shabahang, Judith G. Giri, Alexey I. Nesvizhskii, Alexander A. A. Asea, Anil G. Cashikar, Arundhati Rao, James McLoughlin, Arun Sreekumar

**Affiliations:** 1 Department of Biochemistry and Molecular Biology and Cancer Center, Medical College of Georgia, Augusta, Georgia, United States of America; 2 Center for Molecular Chaperones & Radiobiology, Medical College of Georgia, Augusta, Georgia, United States of America; 3 Department of Pathology, Scott & White Hospital, Temple, Texas, United States of America; 4 Department of Pathology, University of Michigan Medical School, Ann Arbor, Michigan, United States of America; 5 Department of Biostatistics, Medical College of Georgia, Augusta, Georgia, United States of America; 6 Department of Surgical Oncology, Medical College of Georgia, Augusta, Georgia, United States of America; 7 Dan L. Duncan Cancer Center, Baylor College of Medicine, Houston, Texas, United States of America; 8 Illinois Institute of Technology, Chicago, Illinois, United States of America; 9 Tumor Bank Department of Pathology, Medical College of Georgia, Augusta, Georgia, United States of America; Sun Yat-sen University Medical School, China

## Abstract

Pancreatic Adenocarcinoma (PDAC), the fourth highest cause of cancer related deaths in the United States, has the most aggressive presentation resulting in a very short median survival time for the affected patients. Early detection of PDAC is confounded by lack of specific markers that has motivated the use of high throughput molecular approaches to delineate potential biomarkers. To pursue identification of a distinct marker, this study profiled the secretory proteome in 16 PDAC, 2 carcinoma in situ (CIS) and 7 benign patients using label-free mass spectrometry coupled to 1D-SDS-PAGE and Strong Cation-Exchange Chromatography (SCX). A total of 431 proteins were detected of which 56 were found to be significantly elevated in PDAC. Included in this differential set were Parkinson disease autosomal recessive, early onset 7 (PARK 7) and Alpha Synuclein (aSyn), both of which are known to be pathognomonic to Parkinson's disease as well as metabolic enzymes like Purine Nucleoside Phosphorylase (NP) which has been exploited as therapeutic target in cancers. Tissue Microarray analysis confirmed higher expression of aSyn and NP in ductal epithelia of pancreatic tumors compared to benign ducts. Furthermore, extent of both aSyn and NP staining positively correlated with tumor stage and perineural invasion while their intensity of staining correlated with the existence of metastatic lesions in the PDAC tissues. From the biomarker perspective, NP protein levels were higher in PDAC sera and furthermore serum levels of its downstream metabolites guanosine and adenosine were able to distinguish PDAC from benign in an unsupervised hierarchical classification model. Overall, this study for the first time describes elevated levels of aSyn in PDAC as well as highlights the potential of evaluating NP protein expression and levels of its downstream metabolites to develop a multiplex panel for non-invasive detection of PDAC.

## Introduction

Pancreatic ductal adenocarcinoma (PDAC) is the fourth leading cause of cancer death in USA and has one the lowest survival rates for solid cancers [1]. Most patients diagnosed with pancreatic cancer die within 12 months, and only 4% survive 5 years after diagnosis. This is largely due to late presentation by affected patients, thereby making therapeutic intervention difficult [2]. Early diagnosis of pancreatic cancer, including pre-neoplastic lesions (designated as pancreatic intraepithelial neoplasia or PanIN) in average-risk and high-risk patients is desperately needed to improve the survival rate of pancreatic cancer patients [3], [4], [5]. Carbohydrate antigen 19-9 (CA 19-9), also known as sialylated Lewis (a) antigen is the only reliable and widely used biomarker for diagnosis of pancreatic cancer (sensitivity 70%, specificity 87%) [6], [7]; however, its use is largely limited to following the course of disease [8], [9]. It must be noted that CA19-9 is not specific for pancreatic cancer alone, as it is expressed in some other cancers such as cholangiocarcinoma, and benign conditions such as cholangitis and chronic pancreatitis [10], [11], [12], [13]. Recently, several approaches have been used to find candidate biomarker for pancreatic cancer to facilitate early diagnosis including microarrays and proteomics [14], [15], [16], [17], [18], [19], [20], [21], [22], [23].

Proteomic profiling for pancreatic cancer biomarker discovery is still at its early stage; however, the efforts so far have been productive and the results are encouraging [15], [16], [18], [19], [21], [22], [24]. Proteins by virtue of being the functional denominators of cellular phenotype have garnered a lot of attention as potential biomarkers in cancer. Most of the proteomics approaches for PDAC have focused on assessment of tissue proteome [25], [26], [27] and to some extent examination of proteins secreted in the pancreatic juice [18], [22], [23], [28]. The latter constitutes a rich source of cancer-specific proteome contributed by cellular turnover and degradation of highly proliferative cancer cells that are shed into the juice. This has motivated multiple groups to profile the pancreatic juice proteome using both qualitative and quantitative mass spectrometry. These include Surface-Enhanced Laser Desorption/Ionization Time-Of-Flight Mass Spectrometry (SELDI TOF MS) [29], qualitative 2-Dimensional Electrophoresis (2DE)-based mass spectrometry [30] and quantitative Isotope-Coded Affinity Tags (ICAT)-labeled mass spectrometry [18]. Each of these has identified subsets of proteins that are altered in pancreatic cancer compared to non-cancer controls. However, in addition to being limited by the number of patient samples analyzed, none of the secretory proteins identified have been developed further into a clinically testable biomarker format. Here, we report mass spectrometry based proteomic profiling of pancreatic juice specimens from 25 patients (7 benign, 2 carcinoma in situ (CIS) and 16 pancreatic adenocarcinoma or PDAC). The profiling data revealed a set of 56 proteins that were elevated in PDAC patients compared to benign controls (patients diagnosed with pancreatitis, adenoma etc). Among the candidates identified, were proteins previously reported to be elevated in PDAC namely Mucin 1[22], alpha-glycoprotein [31], alpha1-antitrypsin [32], isoforms of 14-3-3 protein, S-100 [33], PARK 7 (autosomal recessive, early onset 7) [34], [35], [36] etc, as well as new ones. The latter included Alpha-Synuclein (aSyn) and metabolic enzymes like Purine Nucleoside Phosphorylase (NP), both of which were elevated in pancreatic juice from patients with PDAC compared to benign controls. Interestingly both aSyn and PARK 7 are known to be pathognomonic to Parkinson's disease (PD) [34], [35], [37], [38], [39]. Further, independent tissue microarray validation for the first time revealed aggregation of aSyn in a subset of PDAC specimens, similar to its presentation in PD. This suggests existence of a novel and thus far unexplored biological mechanism for the development of PDAC, with semblance to PD. Also higher expression of NP coupled to elevated levels of its down-stream metabolites, in serum had the potential to distinguish PDAC patients from benign controls, highlighting their biomarker potential.

## Results

**Figure 1:**
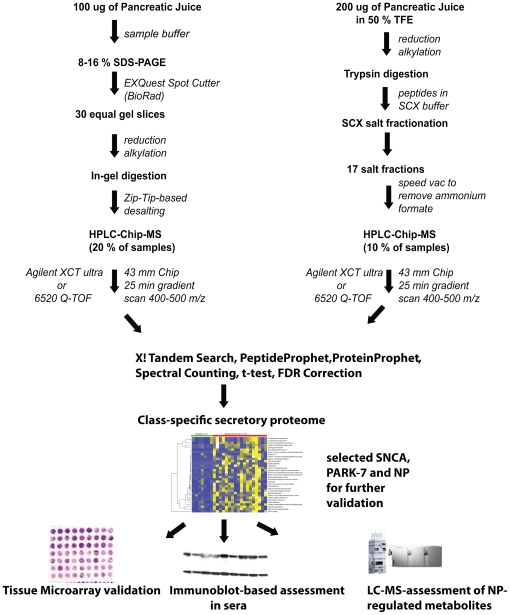
Workflow for Proteomic Profiling of Pancreatic Juice Proteome. Pancreatic juice specimens from 25 patients (7 benign and 18 PDAC) were collected in tubes containing protease inhibitors. The proteome was subjected to pre-fractionation using either SDS-PAGE or SCX-fractionation. For 1D-SDS-PAGE based fractionation, 100 µg of pancreatic juice proteome was electrophoresed on a 8–16% SDS-PAGE and the gel was excised into 30 equal pieces and subjected to in-gel trypsin digestion as described in the methods section. For SCX fractionation, 200 µg of pancreatic juice proteome was first subjected to in-solution trypsin digestion followed by separation of peptides into 20 fractions. Peptides derived from both these methods were separated using reverse phase chromatography on a 43 mm HPLC-Chip and analyzed using an Agilent XCT-ultra ion trap or 6510 QTOF mass spectrometer. The raw spectral files were converted into mzXML format and searched with X!Tandem using a Human IPI-database (see methods for details). The identified peptides and proteins were curated using PeptideProphet and ProteinProphet and the class-specific protein signatures were obtained using coupled to t-test and FDR correction (see methods for details). A subset of PDAC-associated proteins and NP were examined by tissue microarrays and immunblot analysis to confirm their PDAC-specific overexpression in tissues, while expression of NP and its downstream metabolites were examined in serum specimens using immunoblot analysis and targeted LC-MS respectively.

**Figure 1** outlines the strategy employed in this study. In brief, we performed mass spectrometry-based proteome profiling of pancreas-derived juice (n = 25 consisting of 7 benign, 2 CIS, and 16 PDAC juice specimens). Trypsin-digested, fractionated peptides from each clinical specimen were independently identified and quantified using a MudPIT approach using HPLC-Chip nano ESI-MS/MS analysis. The data was normalized on a per sample basis and list of proteins derived from each sample was used to generate a list of pancreas-associated proteome. The proteome was then interrogated for class-specific expression signatures, a subset of which were validated using immunoblot and tissue microarray analysis (TMA) in independent tissues and serum specimens. Furthermore, metabolite levels for NP- regulated metabolites were also examined in pancreas-derived serum specimens. Importantly, our data suggests existence of a Parkinson's disease protein signature in PDAC and describes the combined ability of Purine Nucleoside Phosphorylase protein and its regulated metabolites in serum to distinguish PDAC patients from benign individuals.

### Quantitative Assessment of Pancreatic Juice Proteome

**Figure 2:**
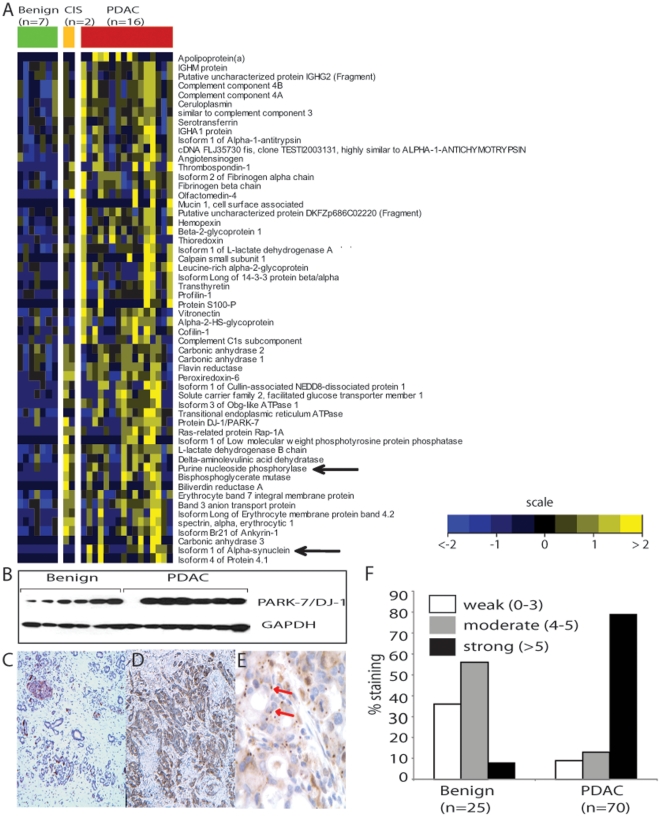
Over view of pancreatic juice proteome and validation of PARK-7 and SNCA expression in PDAC and benign tissues. **A**, Heat map representation of the 56 significantly altered proteins (*P*<0.05 & FDR<12%) in 25 pancreatic juice specimens (7 benign, 2 CIS and 16 PDAC). Columns represent samples and rows refer to proteins. Shades of yellow represent elevated expression of the protein and shades of blue indicate decrease in the protein levels relative to its median expression value (see color scale). **B**, immunoblot confirmation of elevated PARK-7 (DJ-1) expression in PDAC tissues. **C**, representative photomicrograph of the TMA showing immunostaining of aSyn in benign pancreas **D**, same as **C** but in PDAC **E**, Higher magnification of **D** showing aggregated staining pattern for aSyn in PDAC **F**, quantification of TMA staining for aSyn in benign (n = 11) and PDAC samples (n = 67). Magnification of **A** and **B**: 40X, for **C**: 100X.

Using MudPIT proteomic profiling approach 431 proteins were identified with a false discovery rate (FDR) of 1% (**Figure S1 for heat map**); 84% (362) of them were identified in common between benign and PDAC specimens. The total number of spectra from either benign or PDAC specimen were independently normalized, and the normalized cumulative spectral counts of all the peptides for each protein were used as a measure of protein abundance. Based on the statistical analysis of data involving two-sided *t*-test coupled to FDR (false discovery rate) correction [40], proteins were designated as differentially expressed either in the PDAC (up-regulated) or benign (down-regulated) specimens (see Methods section, for details). As shown in **Figure 2A** a total of 56 proteins were found to be significantly (*P*≤0.05, FDR≤12%) up-regulated in pancreatic juice from PDAC patients compared to benign. Included in these were proteins reported earlier to be associated with PDAC as well as additional ones that were found to be elevated in PDAC for the first time in this study. The latter included Alpha-Synuclein (aSyn) that is known to be associated with Parkinson's Disease [37], [38], [39], as well as proteins like Purine Nucleoside Phosphorylase (NP), Valosin Containing Protein (VCP), Thrombospondin-1(THBS1) etc, all of which were found to be elevated in PDAC-derived juice specimens.

### Interrogation of SNCA, PARK-7 and NP in PDAC tissues

aSyn has been known to be an integral component of Lewy body that are characteristic of Parkinson's disease [37]. Further PARK-7 a.k.a. DJ-1 has also been known to be associated with Parkinson's disease and earlier shown to be elevated in pancreatic juice in PDAC patients [31]. However, aSyn overexpression in PDAC has not been reported thus far. We confirmed our profiling data on pancreatic cancer-associated aSyn and PARK 7 overexpression using a combination of TMA and immunoblot analysis performed on tissue specimens from PDAC patients and benign individuals. **Figures 2B** and **S2** validates the elevated expression of PARK-7 in PDAC tissues compared to benign controls. Immunohistochemical staining for PARK-7 (**Figure. S2C**) revealed a heterogeneous pattern of staining of both cytoplasm and nuclei. To correlate PARK-7 expression with various clinical parameters, the staining pattern of the protein in the TMA's were scored both for the intensity and extent by two independent pathologists. The scores for the two parameters were then combined to generate an overall cumulative score which was categorized as weak (0–3), moderate (4–5) or strong (>5). As quantified in **Figure S2C**, about 80% of the PDAC specimens (n = 76) showed strong staining for PARK-7 compared to 65% of the benign tissues (n = 28). Further examination revealed that only 1/28 benign specimens had a strong focal staining (intensity score: 3+) compared to 22/76 PDAC tissues that show such a pattern. Evaluation of staining in the other components including stroma, islets and acini were not performed. Similar validation of aSyn expression in pancreas derived tissues (n = 78) was performed by TMA. **Figure 2 (C–E)** shows a representative photomicrograph of aSyn staining in a benign (panel **C**) and PDAC specimen (panels **D** and **E**). Intense staining for aSyn was seen in epithelial cells of PDAC while benign duct epithelium showed weak staining (refer **Figure S3** for additional photomicrographs of aSyn staining). The staining predominantly exhibited a cytoplasmic distribution with focal peri-apical prominence. Interestingly, in a few of the PDAC tissues wherein the tumor had advanced to a metastatic stage, aSyn expression displayed an aggregated appearance as seen in Parkinson's disease [37] (**Figure 2E**, refer **Figure S4** for additional photomicrographs showing aggregated appearance of aSyn in metastatic PDAC). A semi-quantitative scoring of the TMA as described earlier revealed weak to moderate staining for aSyn in majority (∼92%) of the benign specimens (n = 25) while a large proportion of (∼84%) of the PDAC specimens (n = 70) exhibited moderate to strong staining patterns (**Figure 2F**). Furthermore, the extent of aSyn staining was positively correlated with the T-stage of the disease and existence of perineural invasion (p = 0.018 and 0.0019, respectively, one-sided Spearman's correlation) while it was negatively correlated to the presence of metastatic lesions in the patient (p = 0.0003, **Table S1**). Importantly, the finding of an association for both aSyn and PARK-7 in PDAC progression as revealed in this study suggests a possible nuance in the etiology of pancreatic cancer and Parkinson's disease that needs further interrogation.

**Figure 3:**
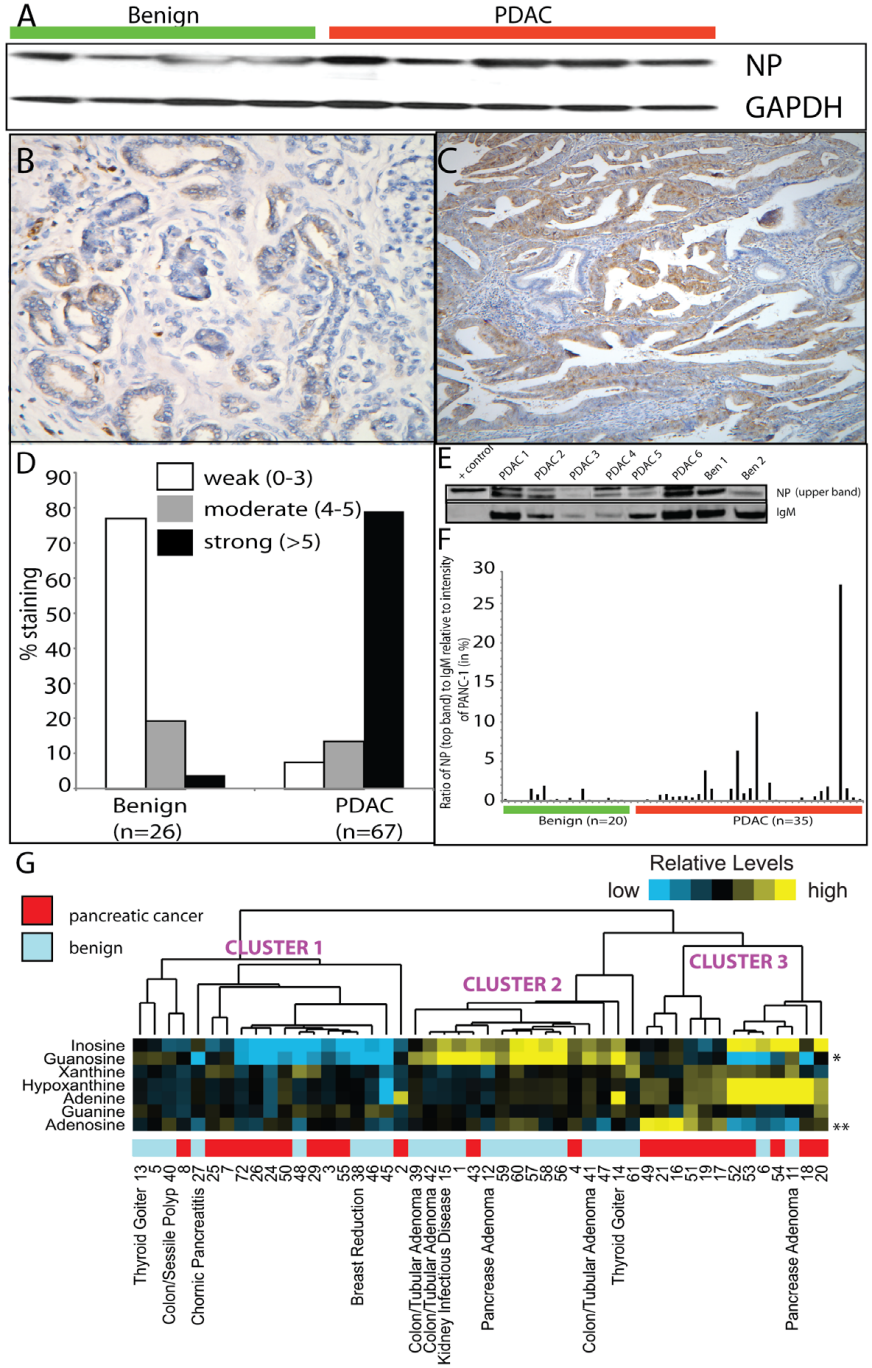
Validation of elevated expression of Nucleoside Phosphorylase (NP) and levels of its regulated metabolites in PDAC. **A**, Immunoblot confirmation of elevated NP expression in PDAC tissues. **B**, representative photomicrograph of the TMA showing immunostaining of NP in benign pancreas **C**, same as **B** but in PDAC **D**, quantification of TMA staining for NP in benign (n = 11) and PDAC samples (n = 49). Magnification of **B** and **C**: 40X **E**, immunoblot showing predominant expression of a doublet at ∼32 KDa immunoreactive to NP antibody in PDAC sera. +control: 10 µg total protein from PANC-1 **F**, immunoblot-based quantification of NP levels in benign and PDAC sera **G**, Unsupervised hierarchical classification of PDAC (n = 24) and benign (n = 24) specimens based on the relative levels of 7 NP regulated metabolites in serum. Columns, samples; rows, metabolites. Asterisks denote metabolites individually correlated (*P*<0.05, *t*-test) with PDAC/benign status.

In addition to the above finding, we also examined the association of NP with PDAC in greater detail as the activity of this enzyme, a critical component of salvage metabolism of purines, is extensively used by tumors to replenish their nucleotide pools. Importantly, as shown in **Figure 2A**, NP levels were higher in PDAC-associated juice compared to benign controls. The higher levels of this protein in PDAC combined with the earlier report on elevated NP activity in cancers [41], motivated us to evaluate this protein in PDAC tissues. As shown in **Figure 3A**, immunoblot analysis confirmed elevated expression of NP in PDAC tissues compared to benign specimens. This was further confirmed using TMA analysis on 67 PDAC and 26 benign samples. NP staining on the TMA was categorized as weak, moderate or strong as described earlier for PARK-7 and aSyn. As shown in **Figure 3B** and quantified in **Figure 3D**, all the benign specimens (n = 26) showed weak (∼77%) to moderate (∼19%) staining for NP while majority of the PDAC specimens (n = 67) exhibited a strong staining pattern (∼79%) for the protein (**Figure 3C, D**). This strong association of NP with PDAC tissue combined with its secretory nature **(** **Figure 2A** **)** prompted us to examine the protein levels for NP in serum of PDAC and control individuals. As shown in **Figure 3E**, immunoblot analysis for NP in serum revealed two bands at ∼32 KDa. Of these the upper band was predominantly seen in PDAC specimens and low to undetectable in majority of the benign sera (**Figure 3E**). When quantified relative to IgM (loading control), the normalized intensity for upper immunoreactive band for NP was found to be significantly elevated in PDAC sera (n = 35, p<0.01, two-sided t-test using log-transformed data) compared to benign controls (n = 20, **Figure 3F**), while it was undetectable in a subset of sera from other tumor types (**Figure S5**). This predominance of NP expression in PDAC sera supported by an earlier report on its elevated enzyme activity in cancers [41] alluded to the possibility of finding altered levels of its target metabolites in serum of PDAC patients. Accordingly, levels of 7 metabolites namely adenine, adenosine, guanine, guanosine, xanthine, hypoxanthine and inosine which are either substrates or products of NP were measured in PDAC and benign sera using targeted LC-MS/MS. Serum levels of each of these metabolites were reported as a ratio to levels of labeled thymine (D_4_-thymine), an internal standard spiked at equimolar levels into each of the serum sample prior to metabolite extraction. The mass spectral data was pre-processed as described in the methods section and the normalized ratio of the metabolites to the internal standard were used to classify the serum samples using unsupervised hierarchical clustering. As shown in **Figure 3G**, the 48 serum samples (24 Benign, 24 PDAC) used in the analysis were delineated into three major clusters based on the relative levels for the 7 metabolites examined, and these significantly demarcated the two sample classes (**Figure 3G**, *p* = 0.00025, one-sided Fisher's exact test). Specifically, 22/24 PDAC specimens were grouped into clusters 1 and 3 respectively while 14/24 benign specimens were grouped in cluster 2 (**Figure 3G**). Furthermore, levels of adenosine and guanosine independently correlated with the PDAC status (*p*<0.04, two-sided t-test).

## Discussion

The treatment of pancreatic adenocarcinoma remains a clinical challenge. The majority of patients are diagnosed at an advanced stage beyond what current therapy can benefit. Most patients have a vague prodrome of symptoms prior to diagnosis making early detection difficult and therefore uncommon [42]. Currently no clinically useful interventions to screen for patients with PDAC are available. The purpose of this project was to evaluate the protein expression profiles of pancreatic juice samples harvested from the pancreatic duct at the time of surgery to potentially identify unique markers that could be used to differentiate benign from malignant pancreatic masses and further delineate different grades of PDAC. 65 proteins were specifically seen only in adenocarcinomas and 4 proteins were observed solely in the benign pancreatic masses. Further, a total of 56 proteins were elevated in pancreatic juice of PDAC compared to benign controls. Of interest, these differentiating profiles revealed the unique presence of proteins associated with Parkinson's disease namely: aSyn and PARK7 [34], [35], [37], [38], [39]. The presence of aSyn in PDAC has not been reported in the literature and previous mouse/drosophila models for this protein have not reported alterations in pancreas function or incidence of pancreatic cancer [43], [44], [45]. However, elevated expression of aSyn has been recently reported in melanoma [46], while its isoform gamma-synuclein (γSyn) a.k.a. Breast Cancer Specific Protein-1 has been shown to be elevated in tumors of breast, uterine, colorectal and pancreas [47], [48], [49], [50], [51], [52], [53], [54]. Further, γSyn that has 63% identity to aSyn at the protein level, has been reported to potentiate invasion in these tumors [49], [50], [51], [53]. In light of this, it was interesting to observe higher levels of aSyn in PDAC. Specifically our tissue arrays demonstrate strong staining for aSyn in tumors compared to moderate to weak staining for benign masses. Of further interest, this protein expressed in a subset of PDAC patients was identified in its aggregated form. This aggregation is characteristic of Lewy Bodies seen in Parkinson's disease which is thought to contribute to the degenerative disease process [37], [55]. Similarly, elevated levels of an additional Parkinson's disease associated protein PARK7 (DJ1) [34], in pancreatic ductal juice of adenocarcinomas corroborate an earlier report [31]. Also other studies have documented DJ-1 over-expression to be associated with moderately differentiated PDAC [56]. Furthermore, DJ-1 has been reported to protect neuronal cells from apoptosis [57], [58] while function as an oncogene in tumor cells [59], [60]. However, the exact mechanism by which PARK-7 exerts its oncogenic effect is not clearly understood, although it is presumed to involve p38 mitogen activated-protein kinase signaling [60]. These results are indicative of a link between PDAC and neurodegenerative disease like Parkinson's disease that will need to be further investigated.

In addition to the above findings, the PDAC-associated secretory proteome also revealed elevated levels of a number of additional metabolic enzymes. One of these was Purine Nucleoside Phosphorylase (NP), the rate limiting enzyme in salvage pathway involving purines, which is operational during inflammation as well as neoplastic progression, and has been highly targeted by various chemotherapeutic agents [61], [62], [63]. In cancers, NP activity has been previously reported to be high in cancer sera [41]. NP expression has also been used to determine the clinical severity of various types of leukemias and lymphomas especially as a ratio to adenosine deaminase (ADA)[64]. Likewise in the area of inflammation, NP deficiency has been reported in immune deficient syndromes, like severe combined immunodeficiency syndrome (SCID) [65], [66], [67]. This strong association of NP with inflammation and its earlier use in assessing severity of leukemia/lymphoma motivated us to evaluate the expression and activity of this protein in PDAC, specifically in patients with antecedent inflammatory conditions like chronic pancreatitis. Furthermore, previous investigations have correlated chronic pancreatitis and the development of pancreatic intraepithelial neoplasia (PanIN) [68]. Thus there are strong correlates to suggest that antecedent chronic inflammation could be a known factor to develop PanIN or PDAC and NP may be a useful marker to monitor the progression from inflammation to PDAC.

Importantly, no previous studies have considered measuring expression of NP protein or its regulated metabolites in PDAC. Our results on NP protein levels in sera show the presence of two bands at ∼32 KDa predominantly in PDAC sera compared to controls. Among these, we presume the differential upper band seen in PDAC sera could represent the phosphorylated form of the protein. Although this needs to be validated in PDAC, NP phosphorylation has been earlier reported in neuronal cells exposed to oxidative stress [69]. Further, in a proof-of-concept setting, our results also show alterations in levels of metabolic intermediates downstream of NP, in PDAC sera, alluding to the existence of an active NP-driven purine salvage pathway in these tumors. This could be an adaptive mechanism developed by PDAC to elude the toxicity of chemotherapeutics as well as the cytotoxic effects of the chronic and intense inflammatory cascade often associated with the tumor; a hypothesis that needs further investigation. Importantly, from a biomarker development perspective, these findings will need to be validated in larger cohort of independent clinical specimens. Furthermore, it will be interesting to focus future investigations on evaluating the potential of NP to predict PDAC's with antecedent chronic pancreatitis and the influence on the development of PanINs in the inflammatory milieu.

In summary, the study describes the secretory proteomic profiles for PDAC which reveals a Parkinson's signature suggestive of a possible nuance between pancreatic tumors and the neurodegenerative disorder. Furthermore, the secretory profiles reveal NP to be elevated in PDAC, indicative of a reliance of the tumor on the salvage pathway. Importantly, levels of NP-regulated metabolites in serum were able to distinguish PDAC from benign patients highlighting its potential for future biomarker development. This finding sets the stage to evaluate NP as a possible marker to predict PDAC with antecedent pancreatitis, a clinical challenge that currently has no predictive markers.

## Materials and Methods

### Subjects and Samples

The study was performed with approval from the Office of Human Research Protection/Institutional Review Board (IRB) of the Scott and White Memorial Hospital, Texas and Medical College of Georgia, Augusta. Written informed consent was obtained from all study subjects. Pancreatic juice specimens were obtained from volunteering PDAC/CIS patients (n = 18) or non-cancer controls (n = 7) undergoing surgical resection of pancreas at Scott and White Memorial Hospital, Texas and Medical College of Georgia Hospital, Augusta, GA. The pancreatic juice samples were collected in tubes containing protease inhibitors and were immediately frozen until further analyses. Detailed clinical and pathology data for this study are available in **Table S2**. For the validation studies tissue microarray consisting of 104 pancreas-derived tissues (n = 28 benign, and 76 PDAC, see **Table S3** for clinical information) were used. These specimens were obtained from Scott and White Memorial Hospital (n = 28), Texas and Medical University of South Carolina (Charleston, SC, n = 50), with approval from the Office of Human Research Protection/Institutional Review Board (IRB) of the respective institutions. Additionally 59 serum samples from 24 PDAC, 23 benign, and 11 cancers of non-pancreatic origin were obtained upon IRB approval from Scott and White Memorial Hospital, Texas and Medical College of Georgia, Augusta and used for biomarker evaluation studies (See **Table S4**).

### Pancreatic juice proteomics using 1D gel protein separation and in-gel digestion

Simple protein separation by 1D-SDS-PAGE before LC-MS/MS (1D-gel-LC-MS/MS) minimizes the sample loss in general, and captures proteins with a wide range of biochemical characteristics (size, extremes of pI, transmembrane domains, PTM's etc) [70], [71], [72], [73]. Pancreatic juice sample recovered from patients with pancreatic cancer and/or pancreatitis/benign tumor (control), containing 100 µg of total protein, were mixed with sample buffer, boiled for 5 min and then separated on 8–16% acrylamide gradient gels (Life-Gels). After staining with SimplyBlue SafeStain (Invitrogen), the individual gel lanes were cut into 30 equal slices using Bio-rad exquest spot cutter. Gel pieces were then subjected to in-gel digestion with trypsin in ammonium bicarbonate (NH_4_HCO_3_) buffer, and peptides extracted as described earlier by Shevchenko et al. [74], with a few minor modifications [75]. Peptides were sequentially extracted in 20 µL 20% formic acid, 100 µL 50 mM NH_4_HCO_3_ and 0.1% trifluoroacetic acid (TFA) in H_2_O:acetonitrile (1∶1) mixture by sonication for 5 min. The extracted peptides were then concentrated in a SpeedVac to ∼10 µL, cleaned and desalted with C_18_ zip-tips. These desalted peptides were completely dried in a SpeedVac and redissolved in 10 µL of 0.1% TFA for LC-MS/MS analysis.

### Fractionation of pancreatic juice proteome using Cation-Exchange Chromatography (SCX)

Pancreatic juice proteins (200 µg) recovered from patients with pancreatic cancer and/or pancreatitis/benign tumor (controls) was first subjected to in-solution double trypsin digestion as described earlier [76], [77]. The resulting peptide mixtures were completely dried in a SpeedVac and resuspended in 40 µL of 0.1% Formic acid in 5% acetonitrile (mobile phase A) and directly loaded onto a 1×150-mm Poly-SEA strong cation exchange column (Michrom Bioresources, Auburn, CA) using Agilent 1200 auto sampler. Buffer containing 1 M ammonium formate, 10% Formic acid in 5% acetonitrile (mobile phase B) was used to create a linear chromatographic gradient at a flow rate of 50 µL/min. A total of 10 fractions were collected over a 40 min gradient encompassing a salt concentration of 0–100 mM ammonium formate. Additional 5 fractions were generated over the next 10 minutes at higher salt concentration range of 100–1000 mM. Fractionated peptides were completely dried and reconstituted in 10 µl of 0.1% TFA prior to LC-MS/MS analysis.

### HPLC-Chip-MS/MS analysis

For mass spectrometry analysis, approximately 10% of peptide fraction was injected into a 1100 Series HPLC-Chip Cube MS interface, and Agilent 6300 Series Ion Trap (XCT ultra) or 6510 QTOF Chip-LC-MS/MS system (all Agilent Technologies). The system is equipped with a HPLC-Chip (Agilent Technologies) that incorporates a 40 nL enrichment column and a 43 mm×75 µm analytical column packed with Zorbax 300SB-C18 5 µm particles. Peptides were loaded onto the enrichment column with 97% solvent A (water with 0.1% formic acid) and 3% solvent B (acetonitrile with 0.1% formic acid) at a flow rate of 4 µL/min. Peptides were then eluted with a gradient 3% to 45% B in 25 min, followed by a steep gradient to 80% B in 5 min at a flow rate of 0.3 µL/min. The total run time, including column reconditioning, was 35 min. MS/MS data was collected as described earlier [76] with minor modification in collision energy for QTOF (slope of 3.7 V/100 Da and offset of 2.5 V). The CID data was analyzed as described below.

### Mass Spectral Data Analysis

MS/MS spectra generated above were extracted from the raw data in mzXML file format using a converter from the Institute for Systems Biology (http://tools.proteomecenter.org/wiki/index.php?title=Software:trapper). The mzXML files were searched using X! Tandem against the Human IPI database version 3.50 (containing 67,665 entries) appended with an equal number of decoy sequences (reversed sequences from the original database). The following search parameters were used to search the spectral files against the database: Tandem/k-score database search, tryptic peptides only, not more than 1 missed cleavage, C+57 as fixed and M+16 as variable modifications respectively and 0.8 Da set as the precursor mass tolerance. In total, 11,297,594 (mostly doubly charged and triply charged) X! Tandem search results were obtained. The search results were further processed using the Trans-Proteomic Pipeline (TPP, www.proteomecenter.org), which includes the PeptideProphet and ProteinProphet tools for peptide and protein-level analysis using default settings. The data set was filtered to keep only proteins identified with a FDR of 1%, resulting in a total of 431 proteins. These were referenced by International Protein Index (IPI) number (http://www.ebi.ac.uk/IPI/IPIhelp.html). Gene Symbol information for the IPI numbers was taken from the EMBL-EBI database for human IPI numbers using http://www.ebi.ac.uk/IPI/IPIhuman.html on March 7, 2008.

### MudPIT analysis

The proteins identified in each sample were grouped and used to generate a composite list for all samples examined in this study. An in-house software was used to resolve ambiguities resulting from protein isoforms and multiple accession numbers [78]. At the beginning, for each protein (or protein group with multiple accession numbers) in the combined lists, the total number of MS/MS spectra that were assigned a peptide mapping to that protein was calculated. Following this, the spectrum count measures for each protein were normalized. Normalization was done to account for small differences in the number of identified peptides across experiments. It was taken as the total number of spectra observed in each experiment that were assigned a peptide from a protein identified in the experiment with high probability. For proteins that were identified in both experiments, the ratio of normalized spectrum counts was calculated and then log-transformed. The resulting distribution was determined using a robust Gaussian distribution fitting procedure with outlier removal (10% of the data), following which the mean and standard deviation (SD) were calculated. Based on the statistical analysis of data involving two-sided t-test coupled to FDR (false discovery rate) correction, proteins were designated either as being up-regulated in PDAC or benign specimens.

### Heat map Plots

Heat maps of proteomic data were drawn using the image function in software R [79]. Heat map in **Figure 2 A**, represents unsupervised hierarchical clustering of the data (rows) grouped by sample type (columns) based on Euclidean distance. Shades of yellow and blue represent an increase and decrease in protein expression respectively relative to the median protein expression calculated post-log transformation and normalization of the data of log-transformed normalized protein levels (see color scale in **Figure 2A**).

For the heat map in **Figure 3 G**, the color coding was derived using the ranking of the log ratio in the distribution of measures for that sample, the intensity of the color being determined by the distance (in standard deviations) from the mean of the distribution. Cluster heat maps (average linkage method) of metabolite data were generated using Cluster.exe and Java Treeview [80], [81], using the log ratios of metabolite versus D4-thymine (internal standard, IS), centered on the median across samples.

### Immunoblot Analysis

Proteins for immunoblotting were resolved by 4–12% NUPAGE gels (Invitrogen) and transferred to PVDF membranes (GE Healthcare Bio-Sciences Corp., Piscataway, NJ). The membranes were blocked for 1 hr with 3% skimmed milk in TBS-T (20 mM Tris.HCl, pH 7.4, 150 mM NaCl, 0.1% Tween 20). Antibodies specific NP-1 and PARK-7 (both monoclonal from Abcam, Cambridge, MA) were added in TBS-T containing 3% skimmed milk and incubated overnight. Antibodies specific to Glyceraldehyde-3-Phosphate Dehydrogenase (GAPDH, Santa Cruz Biotechnology, Santa Cruz, CA) were used to ensure equal protein loading since levels of beta-actin were found to be altered in the pancreatic tissues. The blots were subsequently washed with TBST and incubated with respective secondary antibodies. For LICOR experiments, seablock blocking buffer (ThermoScientific, Rockford, IL) was used instead of 3% skimmed milk. Immunoblot signals were developed using either ECL or ECL-plus chemiluminescence reagent (for tissues, GE Healthcare Bio-Sciences Corp., Piscataway, NJ) or using LICOR for sera. Horse-radish peroxidase labeled or fluorescent labeled antibodies were used for ECL and LICOR (Lincoln, NE) experiments respectively. In experiments involving evaluation of NP levels in serum, 10 µg of total protein from PANC-1 cell line was used in each blot as an internal reference (or + control).

Immunoblot data for NP in PDAC and control serum was quantified using IMAGE J software (http://rsbweb.nih.gov/ij/). Specifically, for each serum the intensity of the upper band for NP and the corresponding IgM band were measured. Following this a ratio of the intensities of the two bands was calculated and represented (as %) relative to the intensity of NP in the internal reference standard (PANC-1).

### Tissue Microarrays

A total of 104 paraffin embedded tissues that included 28 benign pancreas and 76 PDAC were used to construct the tissue microarrays (TMA). TMA's were constructed from representative 3.0 mm cores of tissue (verified by 2 independent pathologists) from cases of adenocarcinomas and pancreatitis. The cores were constructed using a Tissue-Tek Quic-Ray (Sakura, CA) technique and inserted into a pre-formed block, sectioned at 4 micron thickness and subjected to immunohistochemistry. Epitope retrieval was achieved by steam-heating the tissue slides for 20 minutes in high pH (EnVision FLEX Target Retrieval Solution, high pH, DAKO, Carpinteria, CA). Following a 20 minute cooling, the tissue sections were incubated with a 1∶200 dilution of a monoclonal antibody to aSyn from BD Biosciences (San Jose, CA) for 20 minutes. The detection system used was the ENVISION Flex Link (DAKO, Carpinteria, CA) following the manufacturer's instructions. All staining steps were done on the DAKO Autostainer Plus. A similar protocol was used to stain for PARK-7 and NP using their respective primary antibodies (both monoclonal from Abcam, Cambridge, MA).

### Evaluation of PARK-7, aSyn and NP Expression

Immunohistochemical staining was independently evaluated by 2 observers. Staining was scored for extent and intensity. A total score was derived by adding the extent and intensity scores. The extent score represents the estimated percentage of ductal epithelial cells staining positively. The following describes the scores corresponding to various percentages of positively stained ductal epithelial cells: 0, 0%; 1, 1–15%; 2, 16–50%; 3, 51 to 75%; 4, 76 to 100%. The intensity score represents the average intensity of all positive cells such that: score of 0 is considered negative; 1 for weak; 2 for intermediate or moderate and 3 for strong staining intensity. A total staining score was defined as the sum of the intensity and proportion score. Positive staining was defined to be a total or cumulative score greater than 5.

### LC-MS/MS analysis of metabolites regulated by NP

Sample preparation: An aliquot of 150 µL of serum was deproteinized using Amicon® Ultra filter (Millipore) and 100 µl of the filtrate was spiked with 20 µL of the thymine-D4 (0.01 mM, labeled internal standard). The sample was then transferred in to an auto sampler vial, dried at 45°C in a SpeedVac evaporator (Genevac, NY), reconstituted in to 50 µL water:acetonitrile (98∶2) mixture containing 0.1% formic acid and analyzed using Multiple Reaction Monitoring (MRM) on an Agilent 6410 triple quadruple mass spectrometer.

### Multiple Reaction Monitoring (MRM)-based-evaluation of levels of NP-regulated metabolites

MRM for the metabolites was performed using an Agilent triple quadruple mass spectrometer equipped with an Agilent 1200 series binary pump HPLC inlet. Chromatographic separation of metabolites in the filtered serum was performed on a Zorbax Eclipse (Agilent Technologies, CA) XDB-C18 column (50×4.6 mm i.d.; 1.8 µm particle size) maintained at 37°C. The mobile phase used for the gradient-based separation was 0.1% formic acid/0.2% acetic acid/water (v/v/v; A), and 0.1% formic acid/0.2% acetic acid/acetonitrile (v/v/v; B). The initial gradient condition of 2% of B was maintained until first 6 minutes. Following this, the gradient was developed for a total of 6 minutes as follows: 2% B to 30% B and then to 30% B to 90% B, each for 0.5 minutes interval followed by a organic spike from 90% B to 95% B for an additional 5 minutes. The flow rate throughout the separation was maintained at 0.2 mL/min. The samples were kept at 4°C throughout the analysis and the injection volume was kept at 5 µL. The mass spectrometer was operated in the electrospray positive ionization mode with a capillary voltage of 3000 V, a collision gas flow rate of 10 L/min and a nebulizer gas flow rate of 35 L/min. The temperature of nebulizer gas was maintained at 350°C. Nitrogen was used as the collision gas at a collision cell pressure of 2.39×10^−5^ Torr. The details of the MRM transitions for the metabolites examined are given in **Table S5**.

### Statistical analysis of MRM data

Two-sided student t-tests were used to determine relative differences between PDAC and benign samples, using the log ratios of metabolite versus thymine-D_4_ (internal standard, IS); FDR was estimated as *V/R*, where *V* is the expected number of false positives ([nominal p-value]*[number of comparisons made]) and *R* is the number of both true and false positives.

## Supporting Information

Figure S1 **Heat map representation of pancreatic juice proteome.** A total of 431 proteins were detected in 25 pancreatic juice samples (7 benign, 2 carcinoma in situ (CIS), and 16 pancreatic cancer (PDAC). Columns represent samples and rows refer to proteins. Shades of yellow represents elevation of a protein and shades of blue indicates decrease of a protein relative to the median expression value for all the proteins identified.
(TIF)

Figure S2 **Immunostaining for PARK-7 or DJ-1 in pancreatic tissues.** **A**) Representative photomicrograph showing immunostaining of PARK-7 or DJ-1 in benign pancreas **B**) same as **A** but in PDAC, **C**) quantification of TMA staining for PARK-7 in benign (n = 28) and PDAC samples (n = 76).
(TIF)

Figure S3 **Immunostaining of aSyn in pancreatic tissues.** Representative photomicrographs of aSyn staining in a benign (panel **A–C**) and in PDAC specimens (panels **D–F**).
(TIF)

Figure S4 **Representative photomicrographs of the TMA showing aggregated staining pattern for aSyn in PDAC (panels A and B).**
(TIF)

Figure S5 **Comparison of NP expression in PDAC and other cancers.** Immunoblot analysis was performed using NP antibody on serum from PDAC patients (n = 25) and other cancers (n = 11). The latter included serum from breast (n = 2), lung (n = 4), colon (n = 2), kidney (n = 2) and duodenal (n = 1) tumors. In each case the intensities of upper 32 KDa NP immunoreactive protein and 55 KDa IgM (control) were measured using Image-X and the ratio of NP to IgM was derived and plotted on Y-axis.
(TIF)

Table S1 **Spearman's correlation between TMA scores and clinical variables.** The extent of staining for both alpha synuclein and nucleoside phosphorylase were positively correlated with T-stage and perineural invasion status and negatively correlated with presence of metastasis. On the contrary, intensity of alpha synuclein and nucleoside phosphorylase staining were positively correlated with existence of metastasis and negatively correlated with T-stage and perineural invasion. ASN: alpha synuclein, NP: nucleoside phosphorylase, T: T-stage, N: Lymph node status, M: existence of metastasis, Ext: extent of staining, Int: intensity of staining.
(PDF)

Table S2 **Clinical information for pancreatic juice specimens used for mass-spectrometry-based protein profiling.** For columns labeled Node, Metastatic and Margin, a value of 0 indicates absence and 1 indicates presence. Node refers to lymph node metastasis. Evaluation of the Margins were done post-resection of the tumor.
(PDF)

Table S3 **Clinical information of tissues used to construct Tissue Microarrays.** Detailed clinical information was not available for 4 PDAC specimens.
(PDF)

Table S4 **Clinical information of the serum samples used to evaluate NP expression and levels of NP-regulated metabolites.** Asterisk indicate duplicate samples from the same patient.
(PDF)

Table S5 **List of MRM-transitions used to examine the levels of 7 NP-regulated metabolites in serum.** Thymine-D4 is an isotopically-labeled internal standard which was spiked in equimolar amounts into the serum prior to extraction of metabolites.
(PDF)
