# Supplementary figures and images for: Metabolites of Purine Nucleoside Phosphorylase (NP) in Serum Have the Potential to Delineate Pancreatic Adenocarcinoma
Source: PLoS One. 2011 Mar 23;6(3):e17177. doi: 10.1371/journal.pone.0017177 (PMC3063153; doi:10.1371/journal.pone.0017177)

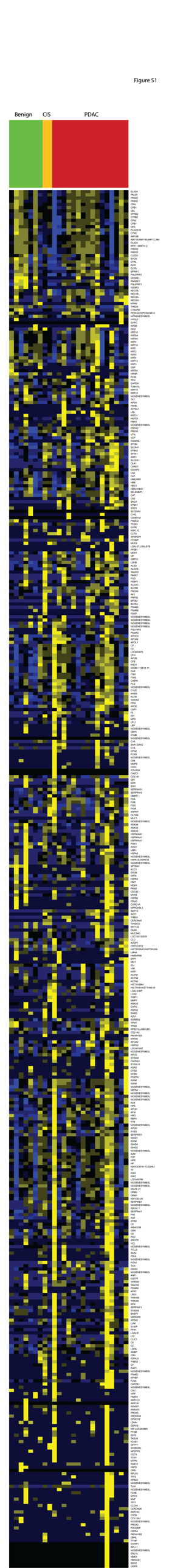

Supplement: Figure S1 — Heat map representation of pancreatic juice proteome. A total of 431 proteins were detected in 25 pancreatic juice samples (7 benign, 2 carcinoma in situ (CIS), and 16 pancreatic cancer (PDAC). Columns represent samples and rows refer to proteins. Shades of yellow represents elevation of a protein and shades of blue indicates decrease of a protein relative to the median expression value for all the proteins identified. (TIF) [file pone.0017177.s001.tif]

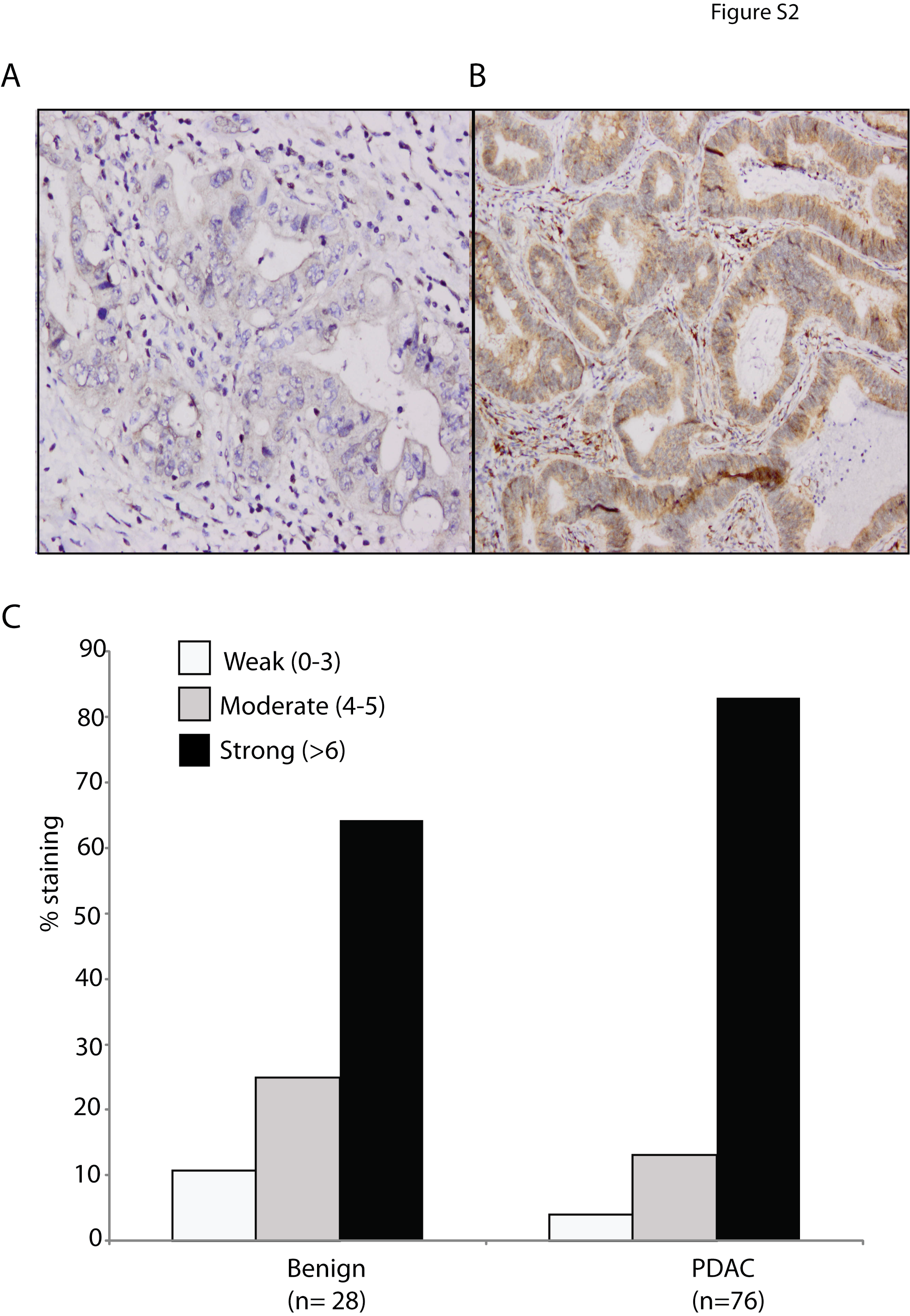

Supplement: Figure S2 — Immunostaining for PARK-7 or DJ-1 in pancreatic tissues. A) Representative photomicrograph showing immunostaining of PARK-7 or DJ-1 in benign pancreas B) same as A but in PDAC, C) quantification of TMA staining for PARK-7 in benign (n = 28) and PDAC samples (n = 76). (TIF) [file pone.0017177.s002.tif]

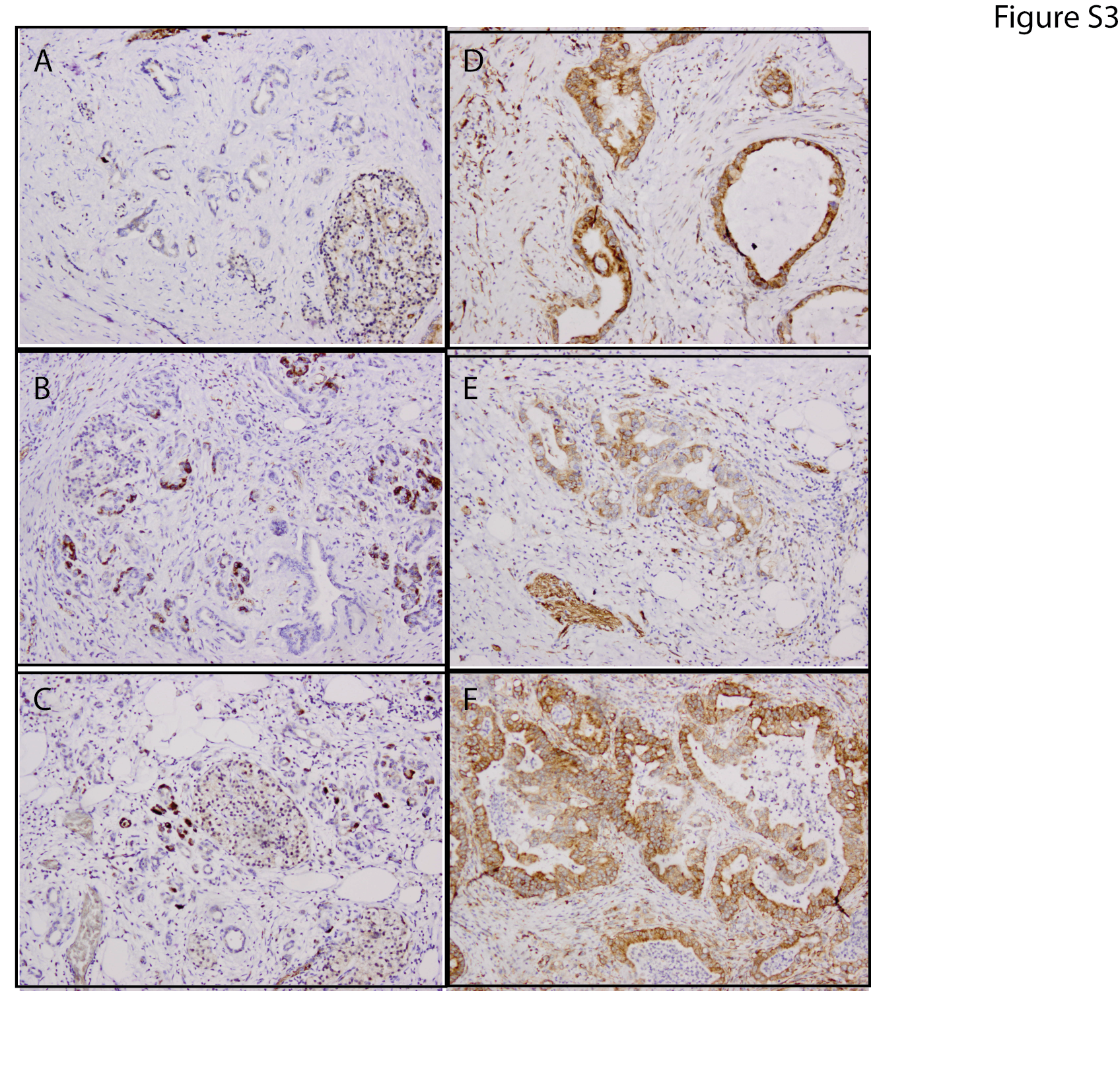

Supplement: Figure S3 — Immunostaining of aSyn in pancreatic tissues. Representative photomicrographs of aSyn staining in a benign (panel A–C) and in PDAC specimens (panels D–F). (TIF) [file pone.0017177.s003.tif]

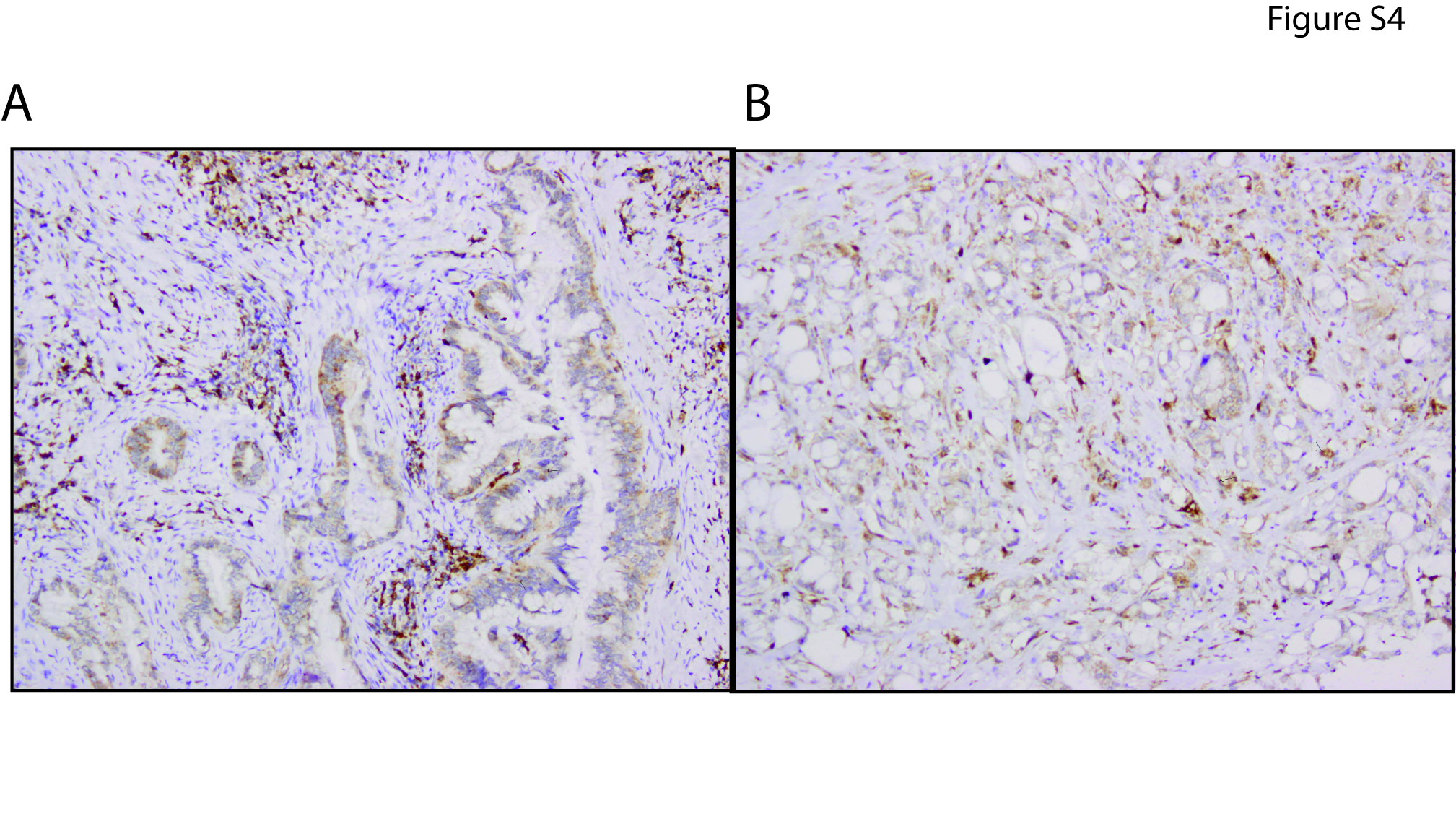

Supplement: Figure S4 — Representative photomicrographs of the TMA showing aggregated staining pattern for aSyn in PDAC (panels A and B). (TIF) [file pone.0017177.s004.tif]

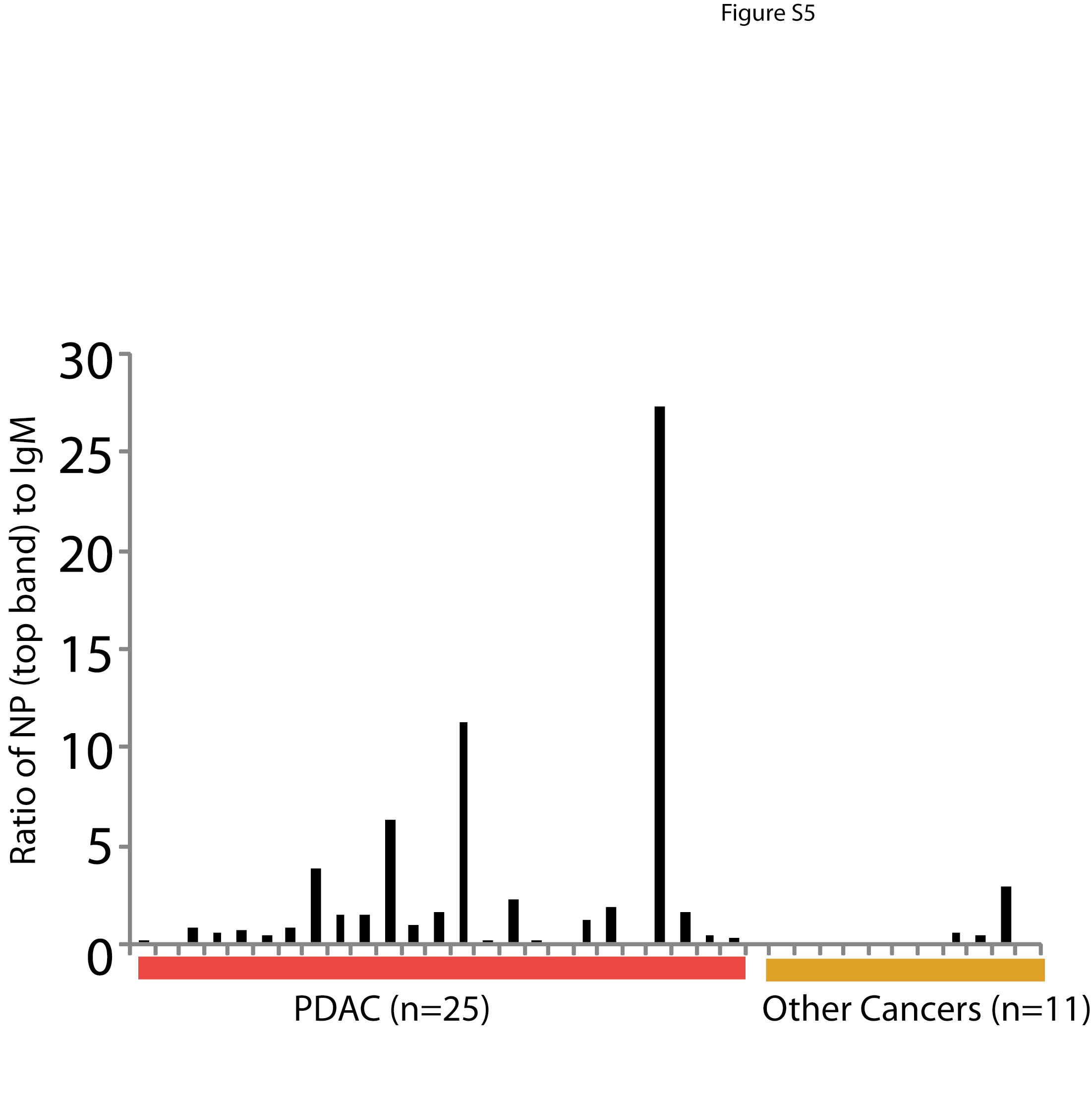

Supplement: Figure S5 — Comparison of NP expression in PDAC and other cancers. Immunoblot analysis was performed using NP antibody on serum from PDAC patients (n = 25) and other cancers (n = 11). The latter included serum from breast (n = 2), lung (n = 4), colon (n = 2), kidney (n = 2) and duodenal (n = 1) tumors. In each case the intensities of upper 32 KDa NP immunoreactive protein and 55 KDa IgM (control) were measured using Image-X and the ratio of NP to IgM was derived and plotted on Y-axis. (TIF) [file pone.0017177.s005.tif]
