# Supplementary material for: Metabolites of Purine Nucleoside Phosphorylase (NP) in Serum Have the Potential to Delineate Pancreatic Adenocarcinoma
Source: PLoS One. 2011 Mar 23;6(3):e17177. doi: 10.1371/journal.pone.0017177 (PMC3063153; doi:10.1371/journal.pone.0017177)
Supplement: Table S1 — Spearman's correlation between TMA scores and clinical variables. The extent of staining for both alpha synuclein and nucleoside phosphorylase were positively correlated with T-stage and perineural invasion status and negatively correlated with presence of metastasis. On the contrary, intensity of alpha synuclein and nucleoside phosphorylase staining were positively correlated with existence of metastasis and negatively correlated with T-stage and perineural invasion. ASN: alpha synuclein, NP: nucleoside phosphorylase, T: T-stage, N: Lymph node status, M: existence of metastasis, Ext: extent of staining, Int: intensity of staining. (PDF) [file pone.0017177.s006.pdf]

Table S1.

	T	N	M	Stage	perineural invasion
**ASN Ext**					
Correlation coefficient	0.250839453	-0.002544603	-0.394146393	-0.242836462	0.340206909
valid cases	70	70	70	69	70
one-sided significance	**0.018107794**	0.491660172	**0.000367649**	0.022188975	**0.001978226**
**ASN Int**					
Correlation coefficient	-0.40970346	-0.049011601	0.42194678	0.108055836	-0.357123491
valid cases	70	70	70	69	70
one-sided significance	**0.000214101**	0.343503778	**0.000137299**	0.188409393	**0.001203216**
**NP Ext**					
Correlation coefficient	0.278095666	-0.018819804	-0.357602341	-0.171840575	0.465412875
valid cases	67	67	67	66	67
one-sided significance	**0.011344735**	0.439924325	**0.001484497**	0.083846774	**3.61646E-05**
**NP Int**					
Correlation coefficient	-0.376321071	-0.23744021	0.388569553	0.071700968	-0.233298566
valid cases	68	68	68	67	68
one-sided significance	**0.000781226**	0.025608226	**0.000529213**	0.282106255	**0.027767052**
