# Supplementary material for: Metabolites of Purine Nucleoside Phosphorylase (NP) in Serum Have the Potential to Delineate Pancreatic Adenocarcinoma
Source: PLoS One. 2011 Mar 23;6(3):e17177. doi: 10.1371/journal.pone.0017177 (PMC3063153; doi:10.1371/journal.pone.0017177)
Supplement: Table S2 — Clinical information for pancreatic juice specimens used for mass-spectrometry-based protein profiling. For columns labeled Node, Metastatic and Margin, a value of 0 indicates absence and 1 indicates presence. Node refers to lymph node metastasis. Evaluation of the Margins were done post-resection of the tumor. (PDF) [file pone.0017177.s007.pdf]

Table S2.

ID	Class membership	Diagnosis	T stage	Stage	Node	Metastatic	Margin	Current Status
PJ2	Benign	Chronic Pancreatitis						
PJ3	Cancer	Ductal Adenocarcinoma		3	1	1	0	Dead
PJ5	Cancer	Ductal Adenocarcinoma	3	3	1	1	0	Dead
PJ6	Cancer	Invasive Carcinoma	1	3	1	0		
PJ7	Benign	Chronic Pancreatitis						
PJ8	Cancer	Duodenal Adenocarcinoma in situ						
PJ9	Benign	Chronic Pancreatitis						
PJ10	Benign	Ampullary Adenoma/Pancreatitis						
PJ11	Cancer	Ductal Adenocarcinoma	3	4	1	1		
PJ12	Cancer	Ampullary Adenocarcinoma	2	2	0	0	0	Alive
PJ13	Cancer	Ductal Adenocarcinoma	3	4	1	1	0	Dead
PJ14	Benign	Pancreatitis						
PJ15	Cancer	Ductal Adenocarcinoma	2	4	0	1	0	
PJ16	Cancer	Ductal Adenocarcinoma	3	3	0	0	0	
PJ17	Cancer	Ductal Adenocarcinoma	2	3	1	0		
PJ18	Cancer	Intraductal Papillary Mucinous Tumor						
PJ19	Cancer	Ampullary Adenocarcinoma	2	2	0	0		Dead
PJ20	Cancer	Ductal Adenocarcinoma	3	4	1	1		Dead
PJ21	Cancer	Invasive Ampullary Carcinoma	2	3	0	0	0	Alive
PJ22	Cancer	Invasive Adenocarcinoma of ampulla of Vater		3				
PJ23	Cancer	Ductal Adenocarcinoma	3	3	1	1	0	Dead
PJ24	Cancer	Ductal Adenocarcinoma	3	3	1	0	0	Alive
PJ25	Benign	Serous Microcystic Adenoma						
PJ26	Cancer	Ductal Adenocarcinoma	2	3	1	1	0	Dead
PJ27	Cancer	Ductal Adenocarcinoma	2	2	0	0	0	Alive
PJ28	Cancer	Ductal Adenocarcinoma	3	3	1	1	0	
