# Supplementary material for: Metabolites of Purine Nucleoside Phosphorylase (NP) in Serum Have the Potential to Delineate Pancreatic Adenocarcinoma
Source: PLoS One. 2011 Mar 23;6(3):e17177. doi: 10.1371/journal.pone.0017177 (PMC3063153; doi:10.1371/journal.pone.0017177)
Supplement: Table S3 — Clinical information of tissues used to construct Tissue Microarrays. Detailed clinical information was not available for 4 PDAC specimens. (PDF) [file pone.0017177.s008.pdf]

Table S3.

Tissue ID	Race/ Ethnicity	T	N	M	Stage	neural invas	Ductal adenocarcinoma	other types of pancreatic carcinoma/ Diagnosis
1	4	4	1	0	3	1	1	
2	4	4	1	0	3	1	1	
3	4	4	1	0	3	1	1	
4	1	4	1	0	3	1	1	
5	4	4	1	0	3	1	1	
6	4	4	1	0	3	1	1	
7	4	3	0	0	1	0		
8	2	4	1	0	3	1	1	
9	4	4	1	0	3	1	1	
10	4	4	0	0		1	1	
11	4	4	1	0	3	1	1	
12	4	4	1	0	3	1	1	
13	4	5	1	0	4	0		ampullary carcinoma
14	4	5	1	0	4	1	1	
15	4	4	0	1	5	1	1	
16	4	2	1	0	3	1	1	
17	4	4	0	0	2	1	1	
18	2	2	1	0	3	1	1	
19	2	4	1	0	3	1	1	
20	4	3	0	0	1	0	1	duodenal carcinoma
21	4	4	1	0	3	1	1	
22	4	4	0	0	2	1	1	cholangio carcinoma
23	4	5	0	0	4	1	1	ampullary carcinoma
24	4	4	1	0	3	1	1	
25	4	4	1	0	3	1	1	mucinous carcinoma
26	4	3	1	0	3	1	1	
27	4	4	0	0	2	0	1	
28	2	4	1	0	3	0	1	
29	4	4	1	0	3	1	1	
30	2	2	0	0	1	1	1	
31	4	2	0	0	1	1	1	
32	4	4	1	0	3	1	1	
33	4	4	1	0	3	1	1	
34	4	4	1	0	3	0	1	ampullary carcinoma
35	2	4	0	0	2	1	1	
36	4	3	0	0	1	0	1	ampullary carcinoma
37	4	4	1	0	3	1	1	
38	4	4	1	0	3	1	1	cholangio carcinoma
39	4	4	0	0	2	1	1	
40	4	5	1	0	4	1	1	
41	4	4	1	0	3	1	1	
42	4	4	0	0	2	1	1	
43	4	4	0	0	2	1	1	
44	4	4	1	0	3	1	1	
45	2	3	1	0	3	1	1	
46	4	3	0	0	1	0	1	ampullary carcinoma
47	4	4	1	0	3	1	1	
48	4	2	0	0	1	1	1	
49	4	4	0	0	2	1	1	ampullary carcinoma
50	4	5	1	0	3	1	1	
51		2	1	0	2	0	1	
52		2	1	0	2	0	1	
53		4	1	0	3	1	1	
54		4	0	0	3	1	1	
55		3	1	1	4	1	1	
56		4	1	1	4	0	1	
57		4	0	0	3	1	1	
58		3	0	1	4	1	1	
59		3	0	0	2	0	1	
60		4	1	1	4	0	1	
61		3	1	0	3	0	1	
62		4	1	1	4	0	1	
63		3	0	0	2	0	1	
64		3	1	1	4	0		ampullary carcinoma
65		2	0	1	4	0		ampullary carcinoma
66		3	1	1	4	0	1	
67		2	0	0	1	0	1	
68		3	1	1	4	1	1	
69		4	0	1	4	1	1	
70		3	1	0	2	1	1	
71		4	0	0	3	0		
72		3	1	0	3	1		
76							0	Pancreatitis
77							0	Pancreatitis
78							0	Pancreatitis
79							0	Pancreatitis
80							0	Pancreatitis
81							0	Pancreatitis
82							0	Pancreatitis
83							0	Pancreatitis
84							0	Pancreatitis
85							0	Pancreatitis
86							0	Pancreatitis
87							0	Pancreatitis
88							0	Pancreatitis
89							0	Pancreatitis
90							0	Pancreatitis
91							0	Pancreatitis
92							0	Pancreatitis
93							0	Pancreatitis
94							0	Pancreatitis
95							0	Pancreatitis
96							0	Pancreatitis
97							0	Pancreatitis
98							0	Pancreatitis
99							0	Pancreatitis
100							0	Pancreatitis
101							0	Pancreatitis
102							0	Pancreatitis
103							0	Pancreatitis
104							0	Pancreatitis
