# Supplementary material for: Metabolites of Purine Nucleoside Phosphorylase (NP) in Serum Have the Potential to Delineate Pancreatic Adenocarcinoma
Source: PLoS One. 2011 Mar 23;6(3):e17177. doi: 10.1371/journal.pone.0017177 (PMC3063153; doi:10.1371/journal.pone.0017177)
Supplement: Table S4 — Clinical information of the serum samples used to evaluate NP expression and levels of NP-regulated metabolites. Asterisk indicate duplicate samples from the same patient. (PDF) [file pone.0017177.s009.pdf]

Table S4.

Sample ID	Diagnosis	Cancer Type	Additional Information
AR1	Adrenal Benign	BENIGN	
AR2*	Pancreatic Cancer	PANCREATIC CANCER	PanIN
AR3	Pancreatic Cancer	PANCREATIC CANCER	High Grade PanIN
AR4	Pancreatic Cancer	PANCREATIC CANCER	
AR5	Pancreatic Cancer	BENIGN	Carcinoma insitu and Pancreatitis
AR6**	Pancreatic Adenoma, Benign	BENIGN	Serous Cyst Adenoma
AR7	Pancreatic Cancer	PANCREATIC CANCER	Chronic Pancreatitis and PanIN
AR8*	Pancreatic Cancer	PANCREATIC CANCER	PanIN
AR9	Kidney Cancer	OTHER CANCER	
AR10	Kidney Cancer	OTHER CANCER	
AR11**	Pancreatic Adenoma, Benign	BENIGN	Serous Cyst Adenoma
AR12	Pancreatic Adenoma, Benign	BENIGN	Serous Cyst Adenoma
AR13	Thyroid Goiter, Benign	BENIGN	
AR14	Thyroid Goiter, Benign	BENIGN	
AR15	Kindney Infectious Disease, Benign	BENIGN	
AR16	Pancreatic Cancer	PANCREATIC CANCER	
AR17	Pancreatic Cancer	PANCREATIC CANCER	
AR18	Pancreatic Cancer	PANCREATIC CANCER	
AR19	Pancreatic Cancer	PANCREATIC CANCER	
AR20	Pancreatic Cancer	PANCREATIC CANCER	
AR21	Pancreatic Cancer	PANCREATIC CANCER	PanIN
AR24	Pancreatic Cancer	PANCREATIC CANCER	
AR25	Pancreatic Cancer	PANCREATIC CANCER	
AR26	Pancreatic Cancer	PANCREATIC CANCER	
AR27	Pancreatitis, Benign	BENIGN	
AR29	Pancreatic/Duodenal Cancer	PANCREATIC CANCER	PanIN
AR30	Breast Cancer	OTHER CANCER	
AR31	Breast Cancer	OTHER CANCER	
AR32	Colon Cancer	OTHER CANCER	
AR33	Colon Cancer	OTHER CANCER	
AR34	Lung Cancer	OTHER CANCER	
AR35	Lung Cancer	OTHER CANCER	
AR36	Lung Cancer	OTHER CANCER	
AR37	Lung Cancer	OTHER CANCER	
AR38	Breast Normal, Benign	BENIGN	
AR39	Colon/Tubular Adenoma, Benign	BENIGN	
AR40	Colon Polyp, Benign	BENIGN	
AR41	Colon/Tubular Adenoma, Benign	BENIGN	
AR42	Colon/Tubular Adenoma, Benign	BENIGN	
AR43	Small Intestine/Pancreatic Cancer	PANCREATIC CANCER	PanIN
AR44	Duodenal Cancer	OTHER CANCER	
AR45	Pancreatic Benign	BENIGN	
AR46	Pancreatic Benign	BENIGN	Follow up shows Hepatocellular Carcinoma
AR47	Pancreatic Benign	BENIGN	
AR48	Pancreatic Benign	BENIGN	
AR49	Pancreatic Cancer	PANCREATIC CANCER	
AR50	Pancreatic Cancer	PANCREATIC CANCER	
AR51	Pancreatic Cancer	PANCREATIC CANCER	
AR52	Pancreatic Cancer	PANCREATIC CANCER	PanIN
AR53	Pancreatic Cancer	PANCREATIC CANCER	
AR54	Pancreatic Cancer	PANCREATIC CANCER	
AR55	Pancreatic Cancer	PANCREATIC CANCER	
AR56	Pancreatic Benign	BENIGN	Earlier history of Cervical Cancer
AR57	Pancreatic Benign	BENIGN	
AR58	Pancreatic Benign	BENIGN	
AR59	Pancreatic Benign	BENIGN	
AR60	Pancreatic Benign	BENIGN	
AR61	Pancreatic Benign	BENIGN	
AR72	Pancreatic Cancer	PANCREATIC Cancer	metastatic
