# Supplementary material for: Metabolites of Purine Nucleoside Phosphorylase (NP) in Serum Have the Potential to Delineate Pancreatic Adenocarcinoma
Source: PLoS One. 2011 Mar 23;6(3):e17177. doi: 10.1371/journal.pone.0017177 (PMC3063153; doi:10.1371/journal.pone.0017177)
Supplement: Table S5 — List of MRM-transitions used to examine the levels of 7 NP-regulated metabolites in serum. Thymine-D4 is an isotopically-labeled internal standard which was spiked in equimolar amounts into the serum prior to extraction of metabolites. (PDF) [file pone.0017177.s010.pdf]

Table S5.

Analyte	Parent ions (m/z)	Product ions(m/z)
Adenine	136.6	119
Adenosine	268.1	136
Guanine	152	135
Guanosine	284.1	152
Xanthine	153	110
Hypoxanthine	137	110
Insoine	269.1	137
Thymine-D4	131	113.9
